# Cytokine Removal in Critically Ill Patients Requiring Surgical Therapy for Infective Endocarditis (RECReATE): An Investigator-initiated Prospective Randomized Controlled Clinical Trial Comparing Two Established Clinical Protocols

**DOI:** 10.1097/MD.0000000000019580

**Published:** 2020-04-10

**Authors:** Fabian Gisler, Thibaud Spinetti, Gabor Erdoes, Markus M. Luedi, Carmen A. Pfortmueller, Anna S. Messmer, Hansjörg Jenni, Lars Englberger, Joerg C. Schefold

**Affiliations:** a Department of Cardiovascular Surgery; b Department of Intensive Care Medicine; c Department of Anaesthesiology and Pain Medicine, Inselspital, Bern University Hospital, University of Bern, Switzerland.

**Keywords:** critical illness, endocarditis, human leukocyte antigen-DR, injury-associated immunosuppression, sepsis

## Abstract

**Introduction:**

Infective endocarditis (IE) and other severe infections induce significant changes in the immune response in a considerable number of affected patients. Numerous IE patients develop a persistent functional immunological phenotype that can best be characterized by a profound anti-inflammation and/ or functional “anergy.” This is pronounced in patients with unresolved infectious foci and was previously referred to as “injury-associated immunosuppression” (IAI). IAI can be assessed by measurement of the monocytic human leukocyte antigen-DR (mHLA-DR) expression, a global functional marker of immune competence. Persistence of IAI is associated with prolonged intensive care unit length of stay, increased secondary infection rates, and death. Immunomodulation to reverse IAI was shown beneficial in early immunostimulatory (randomized controlled) clinical trials.

**Methods:**

Prospective 1:1 randomized controlled clinical study to compare the course of mHLA-DR in patients scheduled for cardiac surgery for IE. Patients will receive either best standard of care plus cytokine adsorption during surgery while on cardiopulmonary bypass (protocol A) versus best standard of care alone, that is, surgery without cytokine adsorption (protocol B). A total of 54 patients will be recruited and randomized. The primary endpoint is a change in quantitative expression of mHLA-DR (antibodies per cell on CD14+ monocytes/ macrophages, assessed using a quantitative standardized assay) from baseline (preoperation [pre-OP], visit 1) to day 1 post-OP (visit 4).

**Discussion:**

This randomized controlled clinical trial (RECReATE) will compare 2 clinical treatment protocols and will investigate whether cytokine adsorption restores monocytic immune competence (reflected by increased mHLA-DR expression) in patients with IE undergoing cardiac surgery.

**Trial registration:**

This protocol was registered in ClinicalTrials.gov, under number NCT03892174, first listed on March 27, 2019.

## 1 Introduction

Infective endocarditis (IE) is a life-threatening clinical condition that is characterized by infection of the endo-(myo)cardium and cardiac valves.^[1]^ IE treatment depends on the severity of the infection and induced tissue damage. As first-line clinical therapy, systemic intravenous antibiotic treatment according to international guidelines is applied.^1^,^2^ In severe IE affecting the function of heart valves, surgical repair and replacement can be deemed necessary. The decision when to operate on a patient is based on internationally approved guidelines^[2]^ and depends on expected or observed complications, severity of valve dysfunction, and size of vegetation(s).^[1]^ The aim of surgery is to control the site of infection and to restore (normal) heart function while repairing or replacing the infected valve and to repair associated defects (abscess). During cardiac surgery itself, the use of a cardiopulmonary bypass circuit is required.

Severe infections, such as IE, are known to induce significant changes in a hosts’ immune response.^3^,^4^,^5^,^6^,^7^ In fact, numerous patients with IE develop a persistent functional immunological phenotype that can best be characterized by a profound anti-inflammation and functional immunological anergy.^3^,^5^,^8^ This seems especially the case in patients with unresolved infectious foci and was previously referred to as “injury-associated immunosuppression” (IAI).^[3]^ IAI can be assessed by measurement of the monocytic human leukocyte antigen-DR (mHLA-DR) expression, a standardized “global” biomarker of immune competence.^8^,^9^ Persistence of IAI is associated with prolonged intensive care unit (ICU) length of stay, increased secondary infection rates, and death.^[3]^

Immunomodulatory therapies aiming to reverse IAI and were shown beneficial in early clinical trials.^10^,^11^ Recent expert panels suggest evaluation of clinical immunomodulatory strategies, including extracorporeal approaches.^[4]^ We could previously show that assessment of the surface expression of HLA-DR on monocytes/macrophages correlates with the functional status of the cellular immune system in critical illness and may be regarded as a global marker of cellular immune function in ICU patients.^3^,^10^,^11^ An intervention to reduce immune mediators may affect the net dysregulated immunological response,^[11]^ that is, to restore functional immunocompetency. Via comparison of 2 clinical protocols (i.e., cardiopulmonary bypass with cytokine adsorption plus best standard of care vs best standard of care alone), we will investigate whether adsorption modulates functional cellular immunocompetence,^[9]^ which was shown of prognostic importance in the postoperative course of respective patients.^[3]^ Cytokine adsorption will be performed during cardiopulmonary bypass with a European Conformity (CE)-marked medical device approved for this indication. Studies have confirmed the safety of this device, but no data on direct immunological effects (i.e., change in net immune status) are available.^11^,^12^,^13^,^14^,^15^,^16^,^17^

We therefore embarked to investigate in a 1:1 randomized controlled clinical trial whether intraoperative cytokine adsorption would improve mHLA-DR expression and thus immune competence.

## 2 Methods

### 2.1 Type of study

Cytokine REmoval in CRitically ill pAtients requiring surgical Therapy for infective Endocarditis (RECReATE) is an investigator-initiated, prospective, single-center randomized controlled clinical trial. Sponsor is Prof. Lars Englberger from the Department of Cardiovascular Surgery of Inselspital, Bern University Hospital. Cytokine adsorption will be performed using a CytoSorb adsorption device (Cytosorb GmbH, Berlin, Germany), a CE-marked and approved medical device for treatment of patients with IE undergoing cardiac surgery. Cytokine adsorption will be employed during surgery for IE on cardiopulmonary bypass only. The study is performed in a tertiary care academic center (University of Bern, Inselspital, Bern, Switzerland) at the Departments of Intensive Care Medicine, Cardiovascular Surgery, and the Department of Anaesthesiology and Pain Medicine. Recruitment started in November 2019 (ongoing) and the actual protocol version is V1.2, dated August 8, 2019.

### 2.2 Approvals/trial registration

This study was approved by the respective competent local ethics committee on human research (Kantonale Ethikkomission, Bern, Switzerland, approval number 201901038). Informed consent will be achieved from all participants/legal representatives. This protocol was registered on March 27, 2019 at clinicaltrials.gov (NCT03892174) before study start. The study is carried out in accordance with the protocol and principles enunciated in the current version of the Declaration of Helsinki, the guidelines of Good Clinical Practice issued by International Council for Harmonization of Technical Requirements for Pharmaceuticals for Human Use (ICH). Results will be published in a peer-reviewed journal and disseminated at international conferences.

### 2.3 Study design

This study is a prospective 1:1 randomized single-blinded (subject blinded) controlled clinical trial including IE patients scheduled for surgery. All patients scheduled for cardiac surgery due to IE are screened for potential study inclusion. Patients willing to participate after having been fully informed about the study by a cardiac surgeon will have to sign written informed consent. Any patient who discontinues the study will be replaced. Study procedures are performed according to the planned time schedule in adherence to visit intervals indicated given in the SPIRIT diagram (Table 1).

**Table 1:**
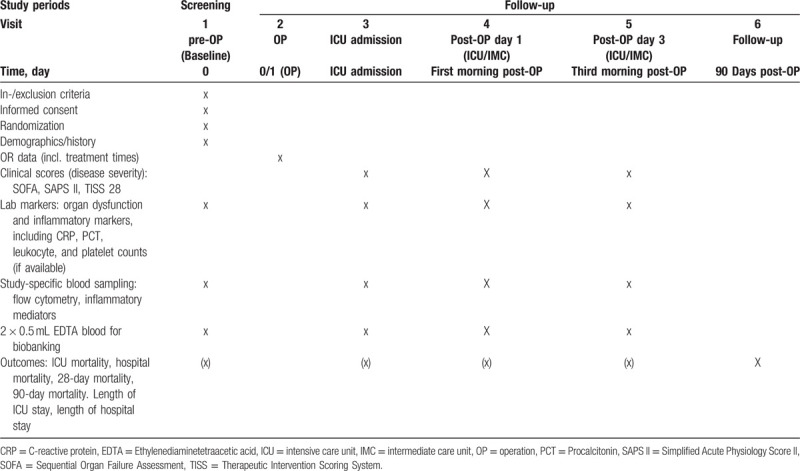
SPIRIT figure indicating visits/study-specific assessments.

### 2.4 Population and sample size calculation

Fifty-four patients undergoing planned surgical treatment for IE will be randomly allocated (1:1 allocation) to receive either best standard of care plus cytokine adsorption (protocol A) or best standard of care alone (protocol B). Allocation to the groups, protocol A or protocol B, is done computerized using the REDCap software. The null hypothesis is that there is no differences in mHLA-DR expression (readout: monoclonal antibodies [mAb/cell]) between the 2 groups. Assuming the response to be normally distributed within each subject group with a standard deviation of 3200 mAb/cell and a true difference in the arms means of 2500 mAb/cell (10,000 vs 7500 mAb/cell of mHLA-DR expression), n = 27 patients will be required to be able to reject the null hypothesis that the population means of the experimental and control groups are equal with probability (power) of 0.8. The type I error probability associated with this test of this null hypothesis is 0.05 (2 sided).

The following inclusion criteria apply:

1. Subjects scheduled for cardiac surgery for infectious endocarditis (according to DUKE criteria) with antibiotic therapy for ≤14 days.
2. Presence of informed consent
3. Age ≥18 years.

The following exclusion criteria apply:

1. Known previous treatment (last 6 months) with immunologically active biologicals or specific immunomodulatory drugs (e.g., rituximab).
2. Known high-dose chronic (i.e., before onset of infectious endocarditis) steroid medication with prednisone equivalent of >30 mg/day.
3. Patients on extracorporeal membrane oxygenation (ECMO), or any other (preoperative) cardiac assist device.
4. Moribund patient (life expectancy <14 days, as judged by investigator).

### 2.5 Outcome measures

#### 2.5.1 Objectives

The purpose of this study is to compare 2 clinical protocols and to investigate whether cytokine adsorption during surgery can modulate the function of monocyte/macrophages, which are key immune cells in patients with severe infections. Monocytic immune competence is assessed by standardized quantitative flow cytometric measurement of mHLA-DR (BD Quantibrite-HLA-DR/Monocyte-BD Biosciences).

#### 2.5.2 Primary endpoint

1. Change in quantitative expression of mHLA-DR (given in antibodies per cell [Ab/cell] on CD14+ monocytes/ macrophages, assessed using a standardized quantitative assay) from baseline (preoperation [pre-OP], visit 1) to day 1 post-OP (visit 4).

#### 2.5.3 Secondary endpoints

1. Change in mHLA-DR from baseline (pre-OP) to post-OP (visit 4) and day 3 (visit 5) post-OP.
2. Area under the curve (AUC) of quantitative mHLA-DR expression between baseline (pre-OP), ICU admission, and days 1 (visit 4) and 3 (visit 5) post-OP.
3. Change in inflammatory markers including cytokines (including white blood count, C-reactive protein, interleukin 6 [IL-6], IL-10, multiplex enzyme-linked immunosorbent assay, inflammatory markers) and immune cell phenotype (flow cytometry) from pre-OP to days 1 (visit 4) and 3 (visit 5) post-OP.
4. Change in organ dysfunction (Sequential Organ Failure Assessment scores including subscores) from baseline (pre-OP) to days 1 (visit 4) and 3 (visit 5) post-OP.
5. Length of ICU/hospital stay (days after ICU admission) and total days in hospital.
6. Cumulative Therapeutic Intervention Scoring System (TISS) points (resource need) while on ICU.
7. ICU-, hospital-, 28-day, 60-day, and 90-day mortality.
8. Total amount of infused fluids on ICU; including number and type of transfusions.
9. Duration of vasoactive drug therapy, cum. vasopressor dose.
10. Length of invasive mechanical ventilation (intubation to extubation), renal function (secondary) ECMO, or other circulatory supporting systems.

### 2.6 Trial monitoring

Trial monitoring is performed according to monitoring plan V 1.0, dated October 31, 2019. The study will be monitored by a trained monitor including 100% of informed consent and inclusion and exclusion criteria data, as well as monitoring of 100% of source data regarding the primary endpoint. Monitoring of trial data and documentation will ensure that protocol requirements, applicable local laws, ICH guidelines, and investigator obligations are fulfilled.

### 2.7 Study-specific analysis and clinical measures

Blood samples for investigating functional cellular immunity, inflammatory response patterns, and course of organ (dys)function will be drawn preoperatively at baseline (visit 1), at ICU admission (visit 3), on postoperative day 1 (visit 4), and on postoperative day 3 (visit 5). In addition, blood samples will be stored for Biobanking (Liquid Biobank Bern). In addition, several clinical outcome parameters, and changes in organ dysfunction and necessity of organ support, will be followed up during hospital stay (Table 1). The primary outcome is measured by flow cytometry using a standardized mHLA-DR assay, as outlined above. ELISA measurements (Cube Dx, St. Valentin, Austria) will be performed using a multiplex ELISA technique. In addition, routine clinical and laboratory data (if available) are used to evaluate and/or to follow-up organ (dys)function(s), inflammation, and/or hemodynamics. Mortality (ICU, hospital, and during follow-up) is evaluated using hospital records, official registries, or other information.

### 2.8 Analysis of results

The primary analysis will be performed on the full data set, that is, the intention-to-treat population. In an additional per-protocol analysis, we will exclude patients with major protocol deviations or missing primary outcome data.

We will apply a linear model corrected for the baseline value to analyze the mean change in quantitative expression of mHLA-DR (mAb/cell on CD14+ monocytes/macrophages) in patients treated with protocol A versus protocol B, namely the following quantity: 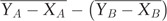, with 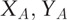 being the protocol A quantitative expression of mHLA-DR at times 1 and 3 respectively and 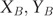 being the protocol B quantitative expression of mHLA-DR at times 1 and 3, respectively, and 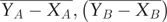 being paired averages. We will assess the normality of model residuals and if the approximate normality is not satisfied we will use transformations of *Y*_*ij*_ and *X*_*ij*_ (with log or Box-Cox transformations). In case we do not achieve normality with any transformation we will use the Wilcoxon-Mann-Whitney test for comparing distributions of 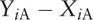 and 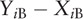.

We will assess the AUC for the quantitative expression of mHLA-DR over time by computing the surfaces under the trajectories of quantitative expression of mHLA-DR and abscissa axis per subject, compute the averages per protocol group, and compare these averages either with an unpaired *t* test (eventually with transformations for normality) or a nonparametric Wilcoxon-Mann-Whitney test.

We will compare the average protocol trajectories of the quantitative expression of mHLA-DR, inflammatory markers, and organ dysfunction by fitting the following mixed-effects model: 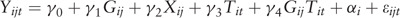 (baseline value is added as a covariate) with *i* being the subject; *j* the protocol; *t* the time-point 2, 3, or 4; *G*_*ij*_ the protocol corresponding to subject *i*; *X*_*ij*_ is the quantitative expression of mHLA-DR at time 1; *T*_*it*_ the time-point 2, 3, or 4 for subject *i*, *ε*_*ijt*_ residuals, and *α*_*i*_ random effect capturing the individual variability of subject *i*. Testing the hypothesis *γ*_4_ = 0 is equivalent to testing the protocol effect over time under assumption of good model fit.

We will provide descriptive statistics for, for example, length of ICU and hospital stay (days after surgical interventions), and cumulative TISS points (resource need) and compare groups by the nonparametric Wilcoxon-Mann-Whitney test. We will show proportions of ICU mortality, hospital mortality, and 28-day mortality and compare groups calculating a risk difference and applying a chi-square test.

## 3 Discussion

Previous data show that functional impairment of cell-mediated immunity reflects an additional “organ” dysfunction in the context of life-threatening injury, such as severe infection.^3^,^5^,^17^ Although large-scale clinical data are missing, IE is known to trigger several humoral and immune cellular pathways, often resulting in a state of immunosuppression in affected individuals, and this was shown of prognostic importance in critically ill patients.^3^,^18^

In the current investigator-driven clinical study RECReATE, we aim to analyze the impact of a cytokine removal strategy (1 treatment session during cardiopulmonary bypass) on key humoral and cellular immune response mechanisms. In brief, we strive to explore whether cytokine adsorption can increase (or even restore) monocytic immune competence (assessed by mHLA-DR expression) in surgically treated patients with IE. Data from previous clinical studies investigating immunostimulatory^[10]^ and selective extracorporeal cytokine removal strategies^[11]^ demonstrate that monocytic function (mHLA-DR-expression) can be restored using comparable measures. Thus, it seems likely that treatment using this alternative cytokine removal strategy will result in increased mHLA-DR expression, reflecting improved monocytic immune function.

Our study has a number of limitations that deserve discussion. First, this is a single-center pilot study with inherent limitations (including limited external validity) set by the chosen study design. Findings in this pilot study should therefore be confirmed in subsequent multicentre investigations. Importantly, however, the primary endpoint is assessed using a standardized quantitative assay with an adequate inter- (and intra-) coefficient of variation shown in multinational comparison studies.^[8]^ Second, although the sample size appears rather limited in this pilot study, it might be considered a strength of the current analysis that the sample size calculation/ power regarding the primary endpoint was partly based on data from respective previous clinical studies. Third, optimal “dose” of the cytokine adsorption applied is currently unknown and, theoretically, one might speculate that the applied “adsorption dose” (treatment only while patients are on cardiopulmonary bypass) might be insufficient. However, this best reflects current practice, in which physicians might chose to prescribe cytokine adsorption based on personal preference.

In conclusion, this pilot trial will answer the important question of whether cytokine adsorption during cardiopulmonary bypass will result in increased mHLA-DR expression, a recognized global functional immune marker. Novel therapeutic approaches seem urgently required in critically ill patients and data from this pilot study will allow to explore new innovative immunomodulatory strategies.

## Acknowledgments

The authors thank all trial physicians, nurses, research nurses, data managers, laboratory staff, and statistical staff for their dedicated support of RECReATE study.

## Author contributions

FG, TS, CAP, and JCS developed the research strategy, initiated the study, drafted and finalized the protocol/ manuscript, applied for EC approval, and supervised the trial. GE, MML, ASM, HJ, and LE helped to draft the manuscript, performed study assessments, recruited patients, and revised the manuscript for important intellectual content. All authors read and approved the final version of the manuscript.

Thibaud Spinetti orcid: 0000-0001-8057-3746.

Joerg C. Schefold orcid: 0000-0001-9162-5724.

Markus Luedi orcid: 0000-0002-9049-2584.

Gabor Erdoes orcid: 0000-0002-6742-1016.

Carmen A. Pfortmueller orcid: 0000-0003-0326-2770.

Anna S. Messmer orcid: 0000-0002-3206-9112.
